# Surgical treatment of severe laryngomalacia: a retrospective study of 11 cases

**DOI:** 10.5935/1808-8694.20130101

**Published:** 2015-10-08

**Authors:** José Antonio Pinto, Henrique Wambier, Elcio Izumi Mizoguchi, Leonardo Marques Gomes, Rodrigo Kohler, Renata Coutinho Ribeiro

**Affiliations:** a Otorhinolaryngologist (Director/Chief).; b ENT Resident - Nucleus of Otorhinolaryngology and Head and Neck Surgery of São Paulo. Nucleus of Otorhinolaryngology and Head and Neck Surgery of São Paulo.

**Keywords:** airway handling, laryngomalacia, respiratory sounds

## Abstract

Laryngomalacia is the most frequent congenital abnormality of the larynx, accounting for approximately 60-75% of congenital stridor cases. Despite its benign and self-limited aspects, 10% of cases require intervention. Currently, supraglottoplasty is considered the standard treatment of severe laryngomalacia.

**Objective:**

To describe the experience of the authors in the surgical treatment of patients with severe laryngomalacia. Methodology: A retrospective study.

**Method:**

The medical records of 11 consecutive cases of severe laryngomalacia who underwent surgical treatment between 2003 and 2012 were analyzed for age, gender, symptoms, associated diseases, surgical technique employed, extubation time, surgical complications, length of hospital stay and clinical outcome.

**Results:**

Of the 11 cases of severe laryngomalacia, six patients (54.5%) were operated with the use of CO_2_ laser and five patients (45.5%) were submitted to the cold technique. Only 1 patient (9.1%) required surgical reintervention. There were no cases of surgical complications. All patients had clinical improvement.

**Conclusion:**

Supraglottoplasty proved to be effective and safe in the treatment of severe laryngomalacia.

## INTRODUCTION

Laryngomalacia is the most common congenital abnormality of the larynx, accounting for about 60% to 75% of congenital stridor cases^1^, ^2^, ^3^. Although its pathogenesis is not fully understood, there is a collapse of supraglottic tissues during inspiration^4^, ^5^, generating a high-frequency inspiratory stridor, exacerbated in the supine position during feeding, agitation and crying^6^, ^7^, ^8^. This stridor usually appears in the first two weeks of life, with an incidence peak around 6 months and spontaneous resolution in 90% of the cases by the second year of life^9^, ^10^. Despite its benign and self-limited nature, 10% of laryngomalacia cases require intervention to relieve the respiratory obstruction^10^, ^11^.

Tracheotomy was considered the safest option for airway maintenance; however, it has become an obsolete method, especially in the last 20 years with the advent of minimally invasive endoscopic techniques. Although some authors advocate hyomandibulopexy as a treatment for severe laryngomalacia, most operations currently involve endoscopic procedures in the supraglottic region, the so-called supraglottoplasties^11^, ^12^, ^13^.

These techniques can be subdivided according to the anatomical region addressed. When the aryepiglottic fold mucosa is excised, we call it: aryepiglottoplasty; likewise, we call it arytenoidoplasty and epiglottoplasty when the supra-arytenoid and epiglottis mucosae are resected, respectively. Other variants described are: epiglottopexy - which corresponds to the lingual-face attachment of the epiglottis to the base of the tongue, and epiglottectomy, which corresponds to the epiglottis cartilage resection^14^.

The definition of severe laryngomalacia is based on the following clinical criteria: apnea, cyanosis, respiratory failure, cor pulmonale, feeding difficulties, low weight and height development, and uncontrollable gastroesophageal reflux - which are severity indicators, being criteria for surgical intervention^10^, ^13^, ^15^, ^16^.

The aim of this study is to describe the experience of the authors in the surgical treatment of patients with severe laryngomalacia evaluated in the pediatric ICU of a tertiary hospital.

## METHOD

Longitudinal historical cohort study, with patient chart review and video recording of 11 surgical patients with severe laryngomalacia, submitted to supraglottoplasty from January 2003 to April 2012.

The charts and video documentation of procedures were obtained from the hospital medical files. We collected data regarding age, gender, symptoms, associated diseases, surgical technique used, extubation time, surgical complications, length of hospital stay and clinical outcome. This study was approved by the Research Ethics Committee, under protocol number 151.689.

## RESULTS

Among the 11 cases evaluated for severe laryngomalacia, nine (81.8%) were males and two were (18.2%) females. All children in the study had stridor and respiratory distress - the main reason for hospitalization; and they were initially evaluated in the intensive care unit.

**Figure 1:**
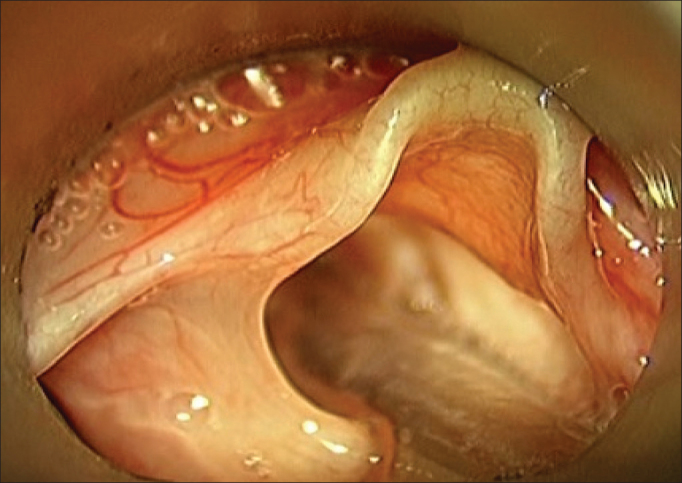
Intraoperative aspect of a 3 - month old child with laryngomalacia, detail of the aryepiglottic fold shortening.

**Figure 2:**
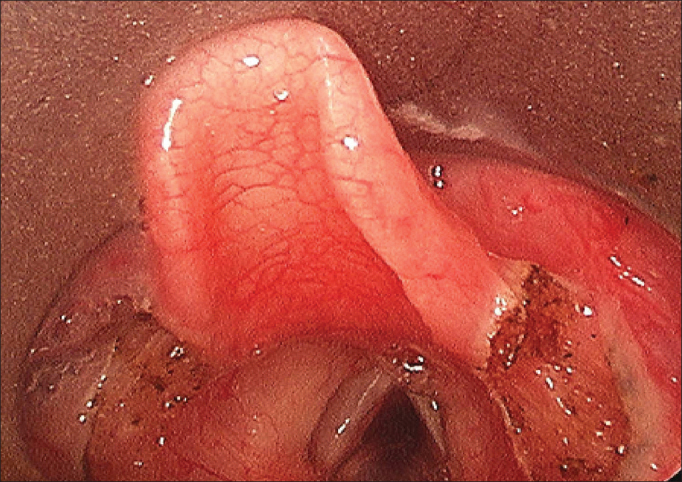
Post - aryepiglottoplasty with CO_2_ laser.

The time of surgery ranged from 1 to 12 months of age. All patients underwent suspension microlaryngoscopy, and supraglottoplasty was performed under general anesthesia and tracheal intubation (Figures 1 and 2 of case number 3).

Postoperatively, all patients were monitored in the intensive care unit. Five patients were extubated after a 48-hour period, four in the immediate post-op and two cases had extubation after 3 and 9 days, respectively.

Only one patient (9.1%) submitted to cold aryepiglottoplasty and extubated in the immediate postoperative period, there was airway obstruction recurrence, which required a new surgical approach, this time using the CO_2_ laser.

There were no cases of surgical complications. All patients had significant stridor and respiratory distress. Data such as age, gender, signs/symptoms, associated disorders, surgery, extubation time, length of hospital stay and clinical outcomes are described on Table 1.

**Table 1:** Results.

Cases	Age	Gender	Signs/Symptoms	Associated diseases	Surgical treatment	Extubation	Hospital stay	Clinical Outcome
1	3 months	M	Stridor, dyspnea, cyanosis	Hypoxic ischemic encephalopathy, renal failure, bronchopulmonary dysplasia	Bilateral aryepiglottoplasty with CO_2_ laser and vaporization of the lingual face of the epiglottis	48h	34 days	Progressive symptom improvement

2	2 months	M	Stridor, dyspnea, cyanosis	No	Bilateral aryepiglottoplasty with the CO_2_ laser	Immediate post-op	1 day	Progressive symptom improvement

3	3 months	F	Stridor, furcula retraction, dyspnea, cyanosis	Hypoxic encephalopathy, ischemic hypoxia	Bilateral aryepiglottoplasty with the CO_2_ laser	48h	7 days	Progressive symptom improvement

4	1 Month	M	Cyanosis, apnea, insufficient weight gain	Gastroesophageal reflux disease (GERD)	Cold bilateral aryepiglottoplasty + epiglottoplasty and removal of the redundant arytenoid mucosa with the CO_2_ laser	48h	35 days	Progressive symptom improvement

5	2 months	M	Dyspnea, bronchospasm	No	1 - Cold bilateral aryepiglottoplasty. 2 – Bilateral aryepiglottoplasty with the CO_2_ laser	Immediate postop. Re-intubation after 3 days for respiratory distress	100 days	Need for reintervention and Progressive symptom improvement

6	2 months	M	Stridor, furcula retraction, intercostal retraction	Down syndrome, GERD	Cold bilateral aryepiglottoplasty	Immediate post-op	20 days	Progressive symptom improvement

7	4 months	M	Dysphagia, Stridor, furcula retraction, intercostal retraction	Interatrial communication	Cold bilateral aryepiglottoplasty	48h	31 days	Progressive symptom improvement

8	10 months	M	Stridor, bronchospasm	None	Cold bilateral aryepiglottoplasty	Immediate postop	12 days	Progressive symptom improvement

9	12 months	F	Respiratory distress	None	Cold bilateral aryepiglottoplasty	72h	20 days	Progressive symptom improvement

10	8 months	M	Dysphagia	GERD	Bilateral Aryepiglottoplasty and CO_2_ laser epiglottoplasty	9 days	35 days	Microaspiration

11	3 months	M	Stridor, Respiratory failure	None	Bilateral Aryepiglottoplasty and CO_2_ laser epiglottoplasty	48h	23 days	Progressive symptom improvement

## DISCUSSION

Consistent with the medical literature, there was a predominance of laryngomalacia cases among male patients^2^, ^17^. The inspiratory stridor was considered the most important symptom of the disease, being present in all patients.^1^, ^6^

In the preoperative classification of laryngomalacia, we rely on clinical signs, i.e., the severity of the disease and not the anatomical classification presented by Holinger & Konior^18^. Most authors indicate the procedure in cases of severe laryngomalacia.^1^, ^2^ Likewise, we indicated the surgical procedure based on a number of factors, including the development delays, feeding issues, severe gastroesophageal reflux, and especially the manifestations resulting from airway obstruction.

Although the results presented in the literature are statistically similar with respect to the use of CO_2_ laser and the cold approach^19^, in our experience the CO_2_ laser offers lower risk of intraoperative bleeding and postoperative edema in cases of greater mucosal resection, as in epiglottoplasty and arytenoidoplasty. Clinical outcome was favorable in all our patients, regardless of the technique and instruments employed. We also emphasize that we have no experience in the treatment of laryngomalacia by the potassium-titanium-phosphate (KTP) laser^20^ or microdebrider^19^, as per suggested by some authors.

The first study to systematically examine the unilateral supraglottoplasty was published in 1995 by Kelly & Gray^21^. The authors had a successful surgery in 94% of cases without major complications in a study involving 18 patients. Only three patients required a bilateral aryepiglottic fold approach. Loke et al.^19^ performed the technique bilaterally in 33 patients, with complete resolution of stridor in 22 cases (68.7%), partial resolution in seven (21.8%), re-operation surgery in two patients; and one patient with multiple malformations underwent tracheostomy. Considering the 19% to 45% rate of surgical revisions described in the literature^4^- with an even higher percentage of unilateral supraglottoplasty cases^8^, we adopt the bilateral aryepiglottoplasty as the procedure of choice at our institution. Thus, the epiglottoplasty and arytenoidoplasty are kept as a complementary method to aryepiglottoplasty, when this alone is not sufficient to improve the supraglottic obstruction.

Complications of supraglottoplasty include bleeding, infection, edema, aspiration, dysphagia, supraglottic stenosis, synechia, respiratory failure and death^2^, ^4^, ^14^, ^22^. Denoyelle et al.^5^ reported a 7.4% complication rate in 136 patients undergoing bilateral supraglottoplasty, and five patients developed supraglottic stenosis. Although this risk has been hypothesized, current studies indicate excessive removal of laryngeal tissues rather than bilateral aryepiglotoplasty^23^ as the main risk factor for the occurrence of complications. No major complication was described in our study.

Schroeder et al.^24^ warned that the risk of liquid aspiration during the pharyngeal phase of swallowing is more common in patients undergoing supraglottoplasty by CO_2_ laser, the main risk factor for the presence of preoperative aspiration. In our study, only one patient underwent aryepiglottoplasty with epiglottoplasty presented with transient aspiration with improvements after the conservative approach.

In a review of 84 cases O'Donnell et al.^25^ concluded that the majority of patients submitted to aryepiglottoplasty to treat laryngomalacia are able to return home after an overnight stay at the hospital. We have not adopted this recommendation, believing it to be prudent monitoring patients in intensive care unit for a minimum of 24 hours, even in cases of extubation immediately after surgery. This provides further support for possible cases of airway obstruction and lower anxiety by relatives.

Toynton et al.^11^ reported an incidence of 47%, and Friedman et al.^26^ reported an incidence of 65% of synchronous airway lesions, especially in patients with severe laryngomalacia undergoing supraglottoplasty, this being one of the possible causes of treatment failure. Although this index is quite considerable, we do not generalize the use of bronchoscopy as a means of diagnosis, being a test of exception. Our diagnosis is based on clinical and nasal-laryngoscopy findings. There were no cases of synchronous lesions in our patients.

Finally, the diagnosis of laryngomalacia is not commonly observed in the general population, being restricted mostly to the tertiary level of care^11^, with spontaneous resolution in most cases^27^, ^28^, ^29^, ^30^. The success rate of supraglottoplasty described in the literature varies from 38% to 100%^4^. In our series, the clinical improvement of all patients submitted to surgical treatment ensured a success rate of 100% of cases.

## CONCLUSION

Supraglottoplasty proved to be an effective procedure in the treatment of respiratory obstruction, enlarging the laryngeal lumen. Due to its high resolution and low complication rate, it is a safe procedure in the treatment of children with severe laryngomalacia, ensuring clinical improvement for the patients.
